# The Epstein-Barr Virus Oncogene EBNA1 Suppresses Natural Killer Cell Responses and Apoptosis Early after Infection of Peripheral B Cells

**DOI:** 10.1128/mBio.02243-21

**Published:** 2021-11-16

**Authors:** Danielle Westhoff Smith, Adityarup Chakravorty, Mitch Hayes, Wolfgang Hammerschmidt, Bill Sugden

**Affiliations:** a Department of Surgery, Medical School, University of Minnesota, Minneapolis, Minnesota, USA; b McArdle Laboratory for Cancer Research, University of Wisconsin–Madison, Madison, Wisconsin, USA; c Research Unit Gene Vectors, Helmholtz Zentrum München, German Research Center for Environmental Health and German Center for Infection Research (DZIF), Munich, Germany; Perelman School of Medicine at the University of Pennsylvania; St. Jude Children's Research Hospital

**Keywords:** B cell, Epstein-Barr virus, ULBP, c-Myc, herpesviruses, innate immunity, natural killer cells

## Abstract

The innate immune system serves as frontline defense against pathogens, such as bacteria and viruses. Natural killer (NK) cells are a part of innate immunity and can both secrete cytokines and directly target cells for lysis. NK cells express several cell surface receptors, including NKG2D, which bind multiple ligands. People with deficiencies in NK cells are often susceptible to uncontrolled infection by herpesviruses, such as Epstein-Barr virus (EBV). Infection with EBV stimulates both innate and adaptive immunity, yet the virus establishes lifelong latent infection in memory B cells. We show that the EBV oncogene EBNA1, previously known to be necessary for maintaining EBV genomes in latently infected cells, also plays an important role in suppressing NK cell responses and cell death in newly infected cells. EBNA1 does so by downregulating the NKG2D ligands ULBP1 and ULBP5 and modulating expression of c-Myc. B cells infected with a derivative of EBV that lacks EBNA1 are more susceptible to NK cell-mediated killing and show increased levels of apoptosis. Thus, EBNA1 performs a previously unappreciated role in reducing immune response and programmed cell death after EBV infection, helping infected cells avoid immune surveillance and apoptosis and thus persist for the lifetime of the host.

## INTRODUCTION

The innate immune system can be activated by both external invaders, including fungi, bacteria, and viruses, and internal stimuli, such as cellular stress or malignancies (1, 2). This system is complex, with one component, natural killer (NK) cells, being able both to secrete immune-activating cytokines, such as gamma interferon and tumor necrosis factor alpha, and to recognize and kill target cells directly (3). NK cells constitute between 5 and 20% of circulating lymphocytes in humans (4) and express several receptors on their surface, including the NKG2D receptor, which can bind to a number of cognate major histocompatibility complex class I (MHC-I)-like ligands (5). NKG2D is an activating molecule, and its binding to ligands can lead to direct killing of the cells expressing those ligands (6). Typically, cells express relatively low levels of NKG2D ligands, but their expression can be upregulated by stress, transformation, or pathogens (7, 8). Members of one family of viruses, the herpesviruses, have been found to regulate individual ligands of the NKG2D family transcriptionally and posttranscriptionally, and patients with NK cell deficiencies often have uncontrolled herpesvirus infections (9–11), highlighting the importance of NK cells in limiting herpesvirus illnesses.

Epstein-Barr virus (EBV) is an oncogenic herpesvirus that infects 90 to 95% of the world’s adult population and causes a variety of B-cell and epithelial cell malignancies in a subset of infected people (12). In tumor cells, EBV is mostly latent, and during latency a variable subset of viral genes is expressed, including minimally one set of viral microRNAs (miRNAs) (BARTs), two noncoding RNAs (the EBERs), and one viral protein, EBNA1 (13). Initial infection with EBV leads to various immune responses, including expansion of CD8^+^ T cells directed against viral antigens and activation of specific subtypes of NK cells (14, 15). Additionally, infection of B cells with EBV can activate the ATR/Chk1 (16–18) and/or the ATM/Chk2 (19) pathway, concomitant with a subset of the infected cells undergoing a period of rapid cell division (17, 19). Activation of these pathways is known to upregulate expression of ligands that can bind the NKG2D receptor and activate NK cells (7). However, in spite of activating various components of the immune system, at least some EBV-positive cells evade the host immune response and survive lifelong in humans.

While EBV has evolved pathways to regulate the host immune response, the majority of viral genes linked to immune evasion or suppression are typically expressed during the lytic or productive phase of the viral life cycle (20). Some lytic genes, such as *BCRF1* and *BNLF2a*, are also expressed early after infection and can inhibit both NK and T cell activity against newly EBV-positive cells (21, 22). However, only a few of EBV’s well-studied latent gene products have been linked to immune evasion or suppression (23–25). The EBV latent protein EBNA1 binds sequence-specifically to both viral and cellular DNA (26–28). EBNA1 mediates maintenance of EBV’s genomes in latently infected cells (29, 30) and can both enhance and inhibit transcription of viral and cellular genes (31–36). The EBNA1 protein contains a long glycine-alanine (Gly-Ala) repeat region that has been linked to inhibiting both its own translation and processing for antigen presentation (37–39). However, no *trans*-acting immune evasion functions have been attributed to EBNA1, which is expressed in all EBV-positive cells. We now show that EBNA1 directly inhibits the expression of both NKG2D ligands and c-Myc by binding cellular DNA near the transcription start sites of these genes. EBNA1’s binding each of these genomic sites enhances the capacity of newly infected cells to evade host immune responses and to survive and proliferate. We have uncovered a vital addition to EBV’s repertoire of immune evasion strategies that allows virus-positive cells to bypass immune responses and persist for the lifetime of the host.

## RESULTS

### EBNA1 directly regulates the expression of the NKG2D ligand ULBP1.

**FIG 1:**
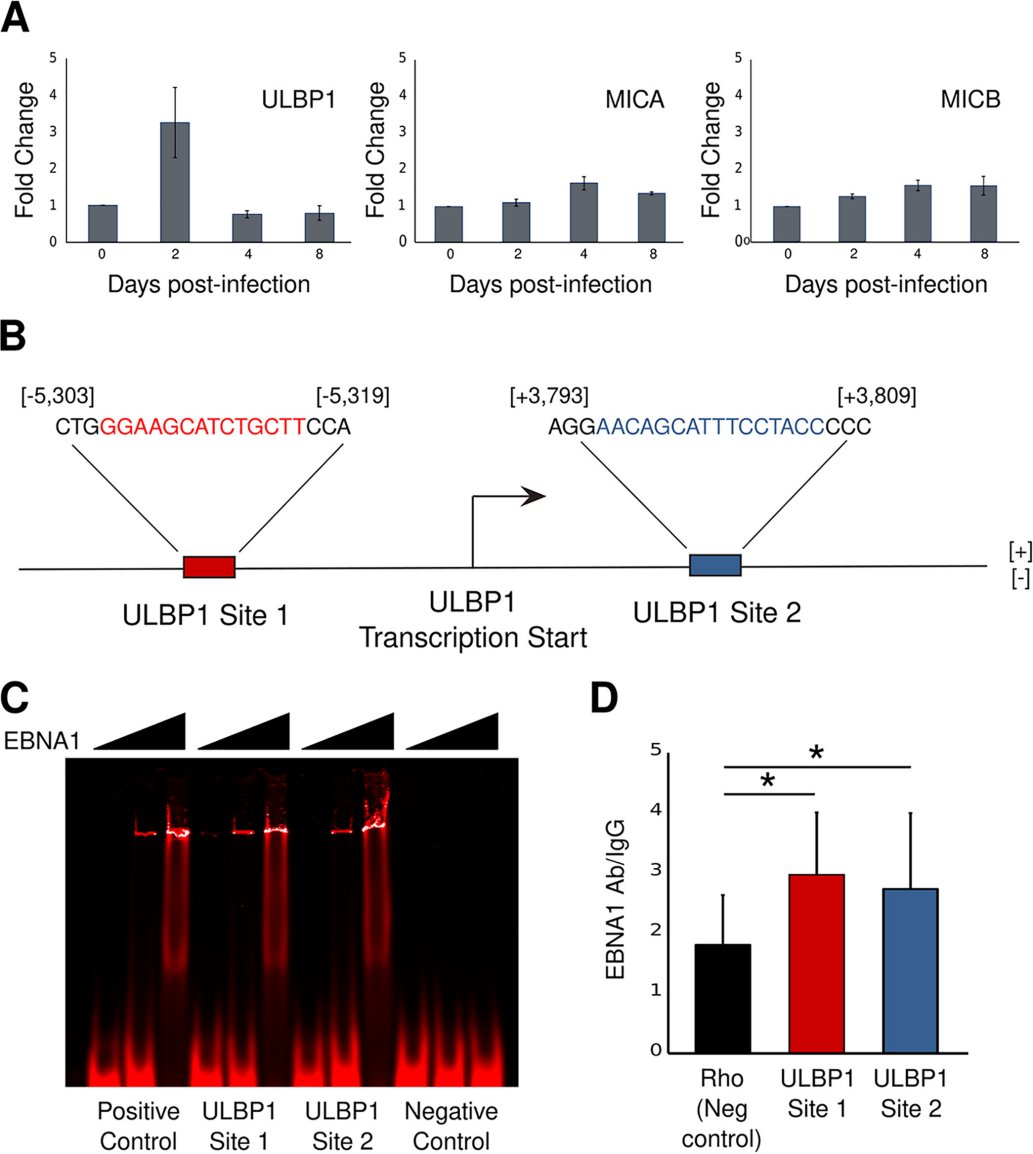
EBNA1 binds cellular DNA near the ULBP1 transcription start site. (A) Levels of ULBP1 increase soon after EBV infection of primary B cells and quickly decline to initial levels. Levels of MICA and MICB do not change significantly. ULBP2 to -6 mRNAs were below the detection threshold (below 25 normalized reads on average). Data are derived from http://ebv-b.helmholtz-muenchen.de/. (B) EBNA1 PWM-predicted binding sites (red and blue sequences) associated with the NKG2D ligand ULBP1 gene. The location of each binding site is given relative to the ULBP1 transcriptional start site. (C) An EMSA showing EBNA1 binding to a positive-control oligonucleotide (EBV Rep* site 1; lanes 1 to 3), oligonucleotides that include ULBP1 site 1 (lanes 4 to 6) or ULBP1 site 2 (lanes 7 to 9), and a negative-control oligonucleotide (a portion of the EBV Raji origin; lanes 10 to 12). For each oligonucleotide, EBNA1 amounts added were none in the left lanes, 1.5 μg EBNA1 in the middle lanes, and 6 μg in the right lanes (*n* = 3; a representative image is shown). (D) Chromatin immunoprecipitation (ChIP)-qPCR measurement of EBNA1 binding to the sites identified in panel A relative to EBNA1 binding to a negative-control region in the genome (proximal to the rhodopsin gene, where there are no expected EBNA1-binding sites) in 721 lymphoblastoid cells. Data shown are relative to IgG binding at each of the sites (*n* = 5; error bars show standard error). *, *P* < 0.05.

When the NKG2D receptor on NK cells binds its ligands, it can activate cytotoxic T cells and/or directly kill the target cells (40, 41). We first screened available transcriptome sequencing (RNA-seq) data (42) to determine the expression patterns of NKG2D ligands in the first 8 days after B cells are infected with EBV *in vitro*. Only the NKG2D ligand ULBP1 was upregulated immediately after EBV infection and subsequently downregulated (Fig. 1A). While we also detected expression of the ligands MICA and MICB, their levels did not change appreciably within the first 8 days after EBV infection (Fig. 1A). We show that downregulation of ULBP1 after the initial increase in its expression after EBV infection is caused at least in part by the EBV latent protein EBNA1 and that this inhibition reduces NK cell-mediated killing of EBV-infected cells. EBNA1 binds many sites in the cellular genome (27, 28, 43) and can both enhance and inhibit transcription of cellular genes (44). We used a position-weighted matrix (PWM; see Fig. S1 in the supplemental material) to search for potential EBNA1-binding sites within the promoter region of the ULBP1 gene and used a position *P* value (described in the work of Dresang et al. [27]) cutoff of 7.7e−5. This is the position *P* value associated with the lowest-affinity EBNA1-binding site in the EBV genome, Rep* site II, to which EBNA1 functionally binds (45). We found two putative EBNA1-binding sites within 5.5 kb of the ULBP1 transcription start site (TSS), one site upstream and one site downstream of the TSS (Fig. 1B). We confirmed that EBNA1 could bind these sequences using electrophoretic mobility shift assays (EMSAs) (Fig. 1C) and also showed that EBNA1 binds these sites *in vivo* in the EBV-positive lymphoblastoid cell line (LCL) 721 using chromatin immunoprecipitation-quantitative PCR (ChIP-qPCR) (Fig. 1D).

10.1128/mBio.02243-21.2 FIG S1 EBNA1 PWM used to predict EBNA1-binding sites near ULBP1, ULBP4, ULBP5, and c-Myc transcription start sites. We used sequences of 73 previously identified EBNA1-binding sites (27) to generate a 16-nucleotide PWM for the binding site of EBNA1 with the online software MEME (http://meme.nbcr.net/meme/). Download FIG S1, DOCX file, 0.3 MB. Copyright © 2021 Westhoff Smith et al. 2021 Westhoff Smith et al. https://creativecommons.org/licenses/by/4.0/ This content is distributed under the terms of the Creative Commons Attribution 4.0 International license.

**FIG 2:**
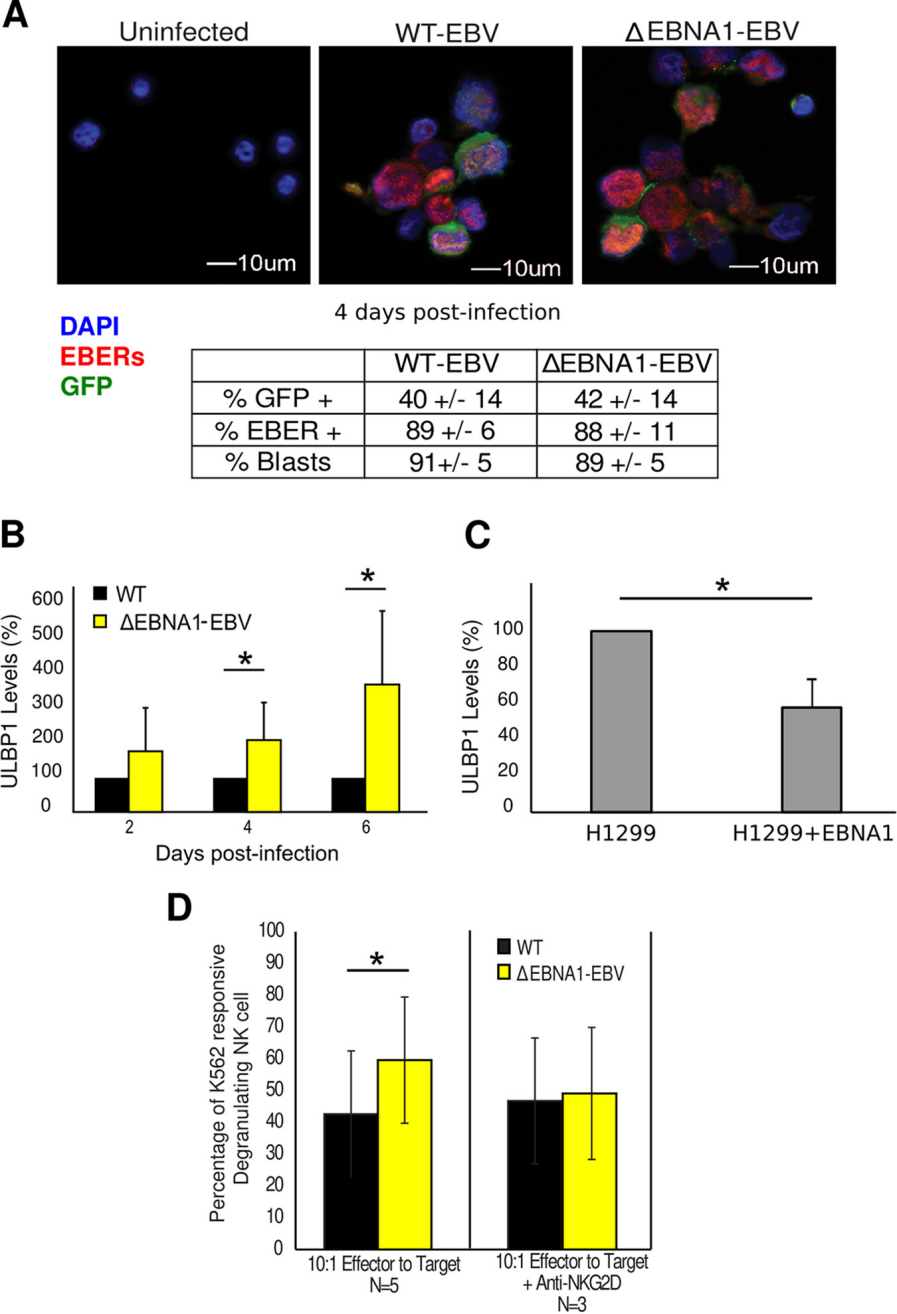
EBNA1 inhibits ULBP1 expression, which protects newly infected cells from NK cell-mediated killing. (A) Primary B cells infected with either wt-EBV or ΔEBNA1-EBV at equal infection rates as determined by EBER *in situ* hybridization (red), GFP expression (green), and blast formation. The mean percentages of cells positive for each marker 4 days postinfection are provided ± standard deviations. At least 50 cells were counted for each condition. (B) Levels of ULBP1 mRNA quantified by qRT-PCR and normalized to TBP expression at different time points following B-cell infection with wt-EBV or ΔEBNA1-EBV. The levels of ULBP1 mRNA were set to 100% for wt-EBV-infected cells (*n* = 3; error bars show standard error). (C) Expression of ULBP1 mRNA in H1299 cells and H1299 cells expressing EBNA1 quantified by qRT-PCR and normalized to GAPDH expression. The levels of ULBP1 mRNA were set to 100% in cells not expressing EBNA1 (*n* = 2; error bars show standard error). (D) NK cell cytotoxicity with B cells 4 days after infection with wt-EBV or ΔEBNA1-EBV (target cells) cocultured at a 1:10 ratio with interleukin-2 (IL-2)-stimulated NK cells (effector cells) in the presence or absence of blocking anti-NKG2D antibody. Data are presented after normalization against uninfected cells and given as the percentage of degranulating cells where response to K562 cells is 100% degranulation (*n* = 5; error bars show standard error). *, *P* < 0.05.

To test whether loss of EBNA1 leads to increased ULBP1 expression in cells infected with EBNA1, we infected primary B cells with either wild-type EBV expressing green fluorescent protein (GFP) (wt-EBV) or a recombinant EBV null for EBNA1-expressing GFP (ΔEBNA1-EBV). Inhibiting EBNA1 in latently infected cells leads over days to the loss of EBV genomes from these cells (46, 47), causing the concomitant loss of expression of other viral genes as well. Therefore, we tested and found that in the first few days after infection with wild-type EBV and ΔEBNA1-EBV, infection dynamics were similar. Primary B cells infected with identical titers (∼1) of these two viruses had similar fractions expressing GFP and were induced to proliferate to similar levels, as measured by B-cell blast formation (Fig. 2A). We also confirmed that equal proportions of cells were infected with virus capable of expressing viral RNAs by performing RNA *in situ* hybridization for the EBV noncoding RNAs, EBERs (Fig. 2A). Importantly, the expression of other EBV latent genes, including EBNA2, EBNA3C, and LMP1, was mostly similar in cells infected with wt-EBV or ΔEBNA1-EBV, although by day 6 postinfection EBNA3C expression had declined (Fig. S2A). However, all the effects of EBNA1 we enumerate—limiting ULBP1 and c-Myc levels as well as reduction of cellular stress responses and apoptosis—are observed by day 4 postinfection when levels of EBNA3C in B cells infected with ΔEBNA1-EBV are statistically the same as the levels seen in B cells infected with wt-EBV.

10.1128/mBio.02243-21.3 FIG S2A At different time points postinfection, we compared mRNA levels of the EBV latent genes LMP1, EBNA2, and EBNA3C by qRT-PCR in cells infected with either ΔEBNA1-EBV or wt-EBV. Levels of the EBV mRNAs were normalized to those of TATA box-binding protein in those cells. The chart shows a ratio of the levels of different EBV mRNAs in cells infected with ΔEBNA1-EBV compared to levels of those mRNAs in cells infected with wt-EBV (*n* = 3; error bars show standard deviation). *, *P* < 0.05. Download FIG S2A, DOCX file, 0.01 MB. Copyright © 2021 Westhoff Smith et al. 2021 Westhoff Smith et al. https://creativecommons.org/licenses/by/4.0/ This content is distributed under the terms of the Creative Commons Attribution 4.0 International license.

When primary B cells were infected with wt-EBV or with ΔEBNA1-EBV, ULBP1 mRNA was induced. However, ULBP1 levels were twice as high 4 days after infection with ΔEBNA1-EBV as in cells infected with wt-EBV (Fig. 2B). By 6 days postinfection, this difference increased to nearly three times more ULBP1 mRNA in primary B cells lacking EBNA1, demonstrating that EBNA1 is required to inhibit the expression of ULBP1 in newly infected B cells. To independently test whether EBNA1 alone is sufficient to decrease ULBP1 mRNA levels, we examined H1299 cells—which express ULBP1 normally—and H1299 cells expressing EBNA1. In the H1299 cells expressing EBNA1, ULBP1 mRNA levels were reduced to less than 60% of the ULBP1 levels in H1299 cells not expressing EBNA1 (Fig. 2C), indicating that EBNA1 is sufficient to inhibit ULBP1 expression. To determine the functional consequences of EBNA1’s inhibition of ULBP1, we tested the susceptibility of B cells infected with wt-EBV or ΔEBNA1-EBV to NK cell-mediated killing. We measured the cytotoxicity of NK cells toward newly infected B cells by the expression of the lysosome-associated membrane protein, CD107a, on the surface of NK cells to determine their degranulation (48). Freshly isolated primary B cells were infected with wt-EBV or with ΔEBNA1-EBV, and 4 days postinfection, when ULBP1 mRNA levels are first significantly higher in cells lacking EBNA1, these newly infected cells were cocultured with autologous primary NK cells (Fig. S2B). EBV sensitizes newly infected B cells to NK cell-mediated cytotoxicity, but in the absence of EBNA1, approximately 40% more NK cells underwent degranulation when cultured with infected B cells than when they were cultured with wt-EBV-infected B cells (Fig. 2D). Importantly, the increased NK cell sensitivity to ΔEBNA1-EBV-infected cells was abrogated in the presence of blocking NKG2D antibody (Fig. 2D), showing the specificity of the assay and confirming that increased levels of ULBP1 RNA detected in these cells correspond to increased levels of the encoded ligand protein. These findings demonstrate that EBNA1 both inhibits ULBP1 expression in EBV-infected B cells and reduces these cells’ susceptibility to NK cell-mediated cytotoxicity; together these functions promote EBV’s evasion of host innate immunity *in vivo*.

10.1128/mBio.02243-21.4 FIG S2B Representative NK cell assay flow cytometry data presenting dot plots of CD56, CD107A-positive (the third quadrant) NK cells that had been cocultured with the target cells as labeled. The percentage of cells within this quadrant is outlined in red. Download FIG S2B, DOCX file, 0.1 MB. Copyright © 2021 Westhoff Smith et al. 2021 Westhoff Smith et al. https://creativecommons.org/licenses/by/4.0/ This content is distributed under the terms of the Creative Commons Attribution 4.0 International license.

### EBNA1 also inhibits the expression of ULBP5, another NKG2D ligand.

**FIG 3:**
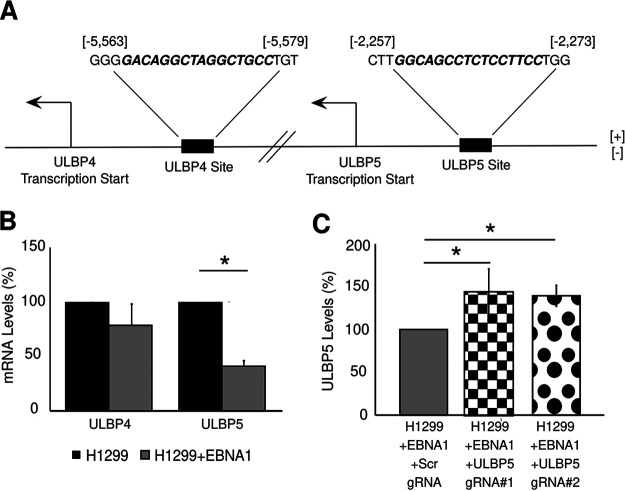
EBNA1 also inhibits the expression of ULBP5, another NKG2D ligand. (A) EBNA1 PWM-predicted binding sites (bold italic sequences) associated with the NKG2D ligand ULBP4 and ULBP5 genes. The location of each binding site is given relative to the ULBP4 and ULBP5 transcriptional start sites. (B) Levels of ULBP4 and ULBP5 mRNA quantified by qRT-PCR and normalized to GAPDH in H1299 cells and H1299 cells expressing EBNA1. The levels of ULBP4 and ULBP5 mRNAs were set to 100% in H1299 cells not expressing EBNA1 (*n* = 3; error bars show standard error). (C) Expression of ULBP5 mRNA in H1299 cells expressing EBNA1 with and without CRISPR-Cas9-induced mutations in the EBNA1-binding site close to the ULBP5 transcriptional start site quantified by qRT-PCR and normalized to GAPDH expression. The level of ULBP5 mRNA was set to 100% in H1299 cells expressing EBNA1 without any CRISPR-Cas9-induced mutations (*n* = 3; error bars show standard error). *, *P* < 0.05.

EBV infects both B cells and epithelial cells *in vivo*, and some NKG2D ligands are expressed primarily on cells of epithelial origin. For example, ULBP4 is expressed mainly in cells derived from the ectoderm, including skin cells and cells lining the mouth and esophagus (49, 50), and ULBP5 mRNA was detected only in cells of epithelial or endocrine origin (51). We used the PWM noted earlier to scan for EBNA1-binding sites near the TSS of other ULBPs and found such sites for ULBP4 and ULBP5 (Fig. 3A). Expression of ULBP4 and ULBP5 genes, which are endogenously expressed in normal oral keratinocytes (NOKs), was found to decrease 3-fold in the presence of the Akata strain of EBV (NOKs-Akata) (Fig. S3A). However, NOKs-Akata cells maintain a latency type I/II pattern of EBV gene expression in which both EBNA1 and LMP2A are expressed (52). To test specifically whether EBNA1 alone can reduce the expression of ULBP4 and ULBP5, we generated H1299 cells expressing full-length EBNA1. In H1299 cells expressing EBNA1, ULBP5—but not ULBP4—levels were reduced significantly (Fig. 3B), making it likely that EBNA1 can inhibit ULBP5 but not ULBP4 expression. To establish that EBNA1 directly inhibits ULBP5 expression by binding the predicted site near the ULBP5 TSS, we used CRISPR-Cas9 to mutate this EBNA1-binding site (Fig. S3B). In two H1299 clones expressing EBNA1 and with confirmed mutations at the EBNA1-binding site, levels of ULBP5 mRNA increased significantly compared to an H1299 clone expressing EBNA1 and with an intact EBNA1-binding site (Fig. 3C). This finding demonstrates that abrogating EBNA1 binding near the TSS of the ULBP5 gene increases its expression, further underscoring that EBNA1 directly inhibits expression of NKG2D ligands through its DNA binding activity.

10.1128/mBio.02243-21.5 FIG S3A Expression of ULBP4 and UBLP5 mRNA in NOKs cells in the presence or absence of EBV quantified by qRT-PCR and normalized to GAPDH expression (*n* = 3; error bars show standard error). Download FIG S3A, DOCX file, 0.02 MB. Copyright © 2021 Westhoff Smith et al. 2021 Westhoff Smith et al. https://creativecommons.org/licenses/by/4.0/ This content is distributed under the terms of the Creative Commons Attribution 4.0 International license.

10.1128/mBio.02243-21.6 FIG S3B Mutations in the EBNA1-binding site near the ULBP5 transcription start site introduced by CRISPR-Cas9 mutagenesis. Sequences from H1299 clones—5-3 and 5-9—are shown here. The sequence in red shows the EBNA1-binding site; red dashes indicate deletions, and blue text indicates insertions. Download FIG S3B DOCX file, 0.03 MB. Copyright © 2021 Westhoff Smith et al. 2021 Westhoff Smith et al. https://creativecommons.org/licenses/by/4.0/ This content is distributed under the terms of the Creative Commons Attribution 4.0 International license.

### EBNA1 attenuates γH2AX levels and apoptosis after EBV infection of B cells.

**FIG 4:**
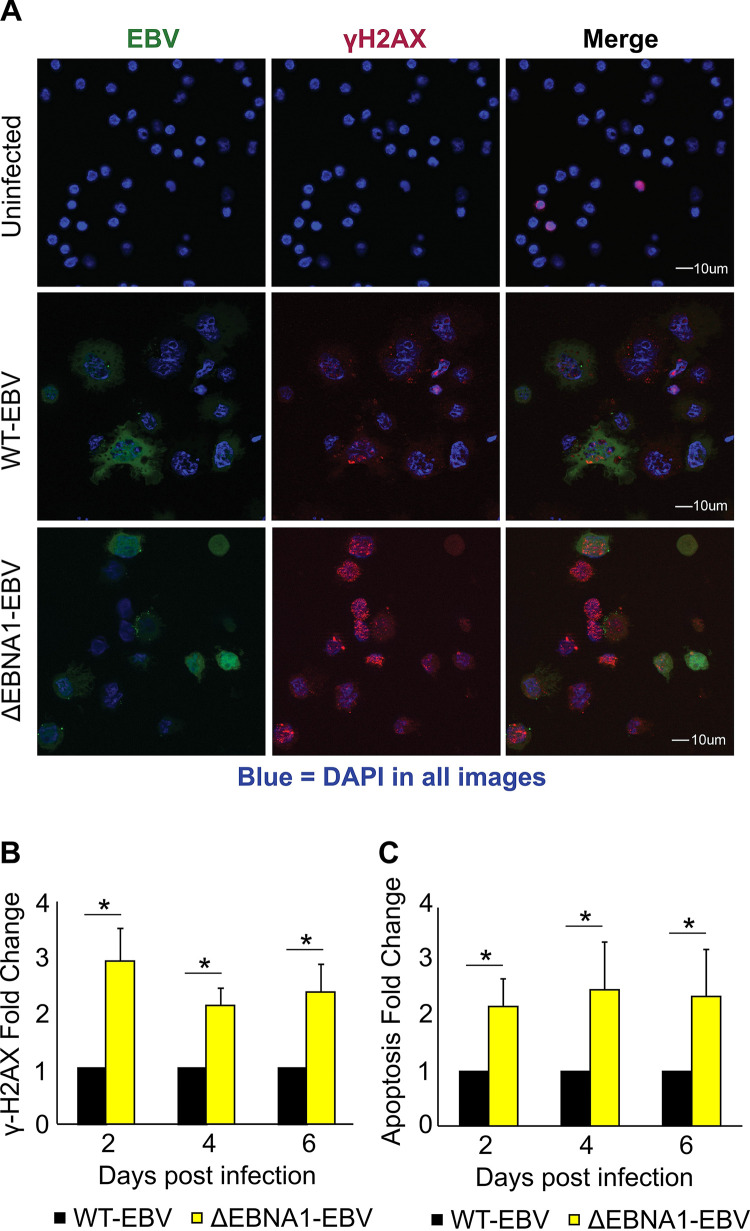
EBNA1 attenuates cellular stress and apoptosis after EBV infection of B cells. (A) Representative immunofluorescence images of γH2AX foci (red) in uninfected primary B cells or B cells 4 days after infection with wt-EBV or ΔEBNA1-EBV. Green indicates GFP expression from either the recombinant wt-EBV or ΔEBNA1-EBV. (B) Fold enrichment of γH2AX focus-positive B cells infected with ΔEBNA1-EBV relative to cells infected with wt-EBV at 2, 4, and 6 days postinfection (*n* = 5; error bars show standard error). (C) Fold enrichment of Apologix-identified caspase-positive B cells infected with ΔEBNA1-EBV relative to cells infected with wt-EBV at 2, 4, and 6 days postinfection (*n* = 5; error bars show standard error). At least 50 cells were counted for each condition. *, *P* < 0.05.

Expression of NKG2D ligands can also increase due to cellular stress responses, such as activation of the ATR/Chk1 and ATM/Chk2 pathways, which play vital roles connected to DNA damage and replication stress (7, 8). B cells infected with EBV have been reported to activate these stress-related pathways early after infection (16–19), although more recent evidence indicates that EBV infection of B cells primarily activates the ATR/Chk1 pathway (16–18). Phosphorylation of the histone variant H2AX—forming γH2AX—is an acknowledged marker of DNA damage and cellular stress (53). To test whether EBNA1 plays a role in limiting phosphorylation of H2AX, we infected freshly isolated, primary B cells with wt-EBV and ΔEBNA1-EBV and stained for γH2AX and for apoptotic cells using an Apologix apoptosis assay (Fig. 4A). The fraction of newly infected cells positive for γH2AX more than doubled by 2 days postinfection in the absence of EBNA1, and this increase continued to be present several days postinfection (Fig. 4A and B). Likewise, the fraction of newly infected cells that exhibited active caspases more than doubled 2 days after infection with ΔEBNA1-EBV when compared to cells infected with wt-EBV (Fig. 4C). These findings show that EBNA1 reduces cellular stress responses and apoptosis, thereby promoting the survival of B cells newly infected with EBV. Furthermore, because cellular stress can increase expression of NKG2D ligands, by attenuating these phenomena, EBNA1 also likely limits expression of these ligands.

### EBNA1 attenuates c-Myc levels in newly infected B cells.

**FIG 5:**
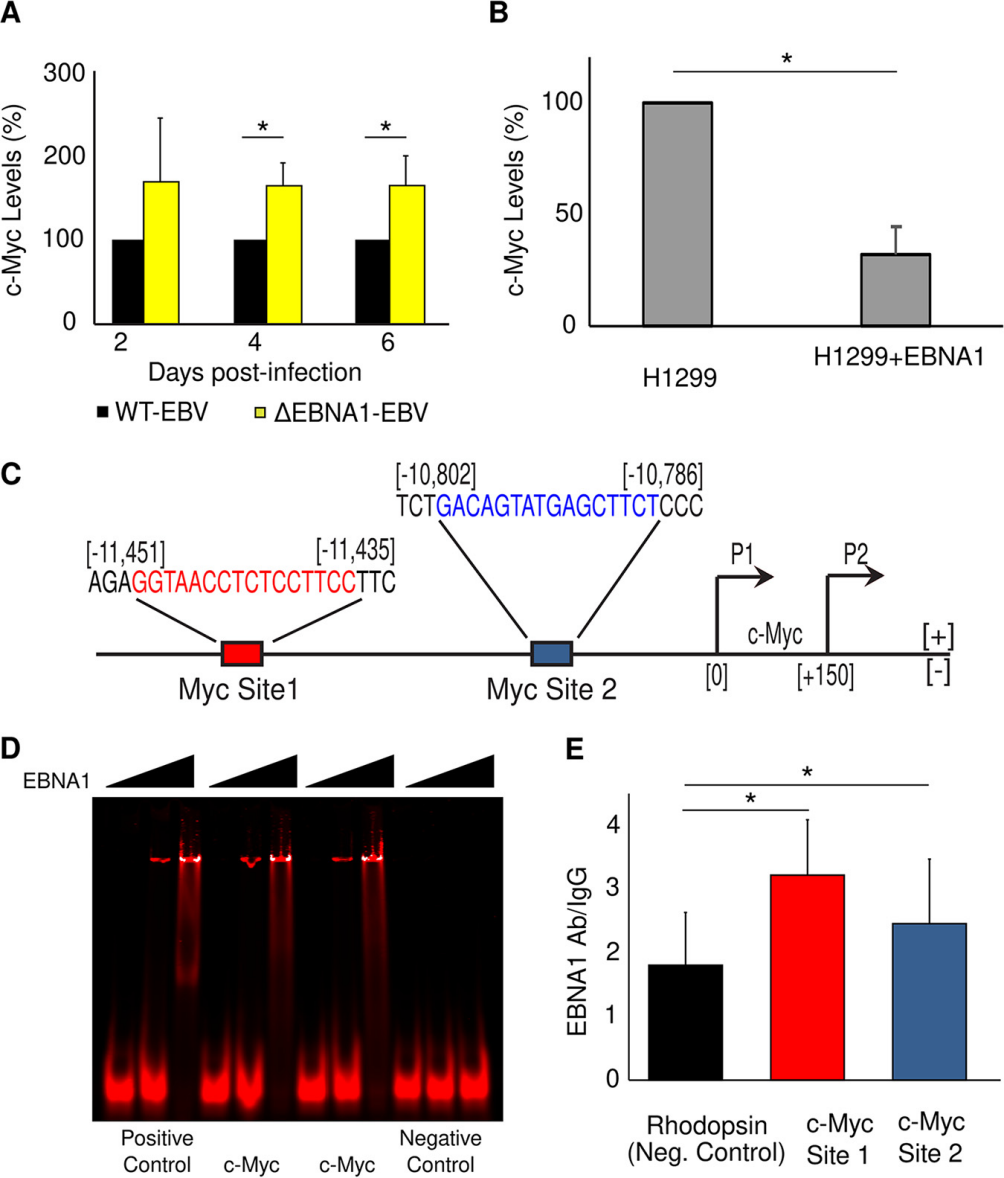
EBNA1 inhibits expression of c-Myc. (A) Levels of c-Myc mRNA quantified by qRT-PCR and normalized to TBP expression at different time points following B-cell infection with wt-EBV or ΔEBNA1-EBV. The levels of c-Myc mRNA were set to 100% for wt-EBV-infected cells (*n* = 5; error bars show standard error). (B) Expression of c-Myc mRNA in H1299 cells and H1299 cells expressing EBNA1 quantified by qRT-PCR and normalized to GAPDH expression. The level of c-Myc mRNA was set to 100% for cells not expressing EBNA1 (*n* = 2; error bars show standard error). (C) EBNA1 PWM-predicted binding sites (red and blue sequences) associated with the c-Myc gene. The location of each binding site is given relative to the c-Myc transcriptional start site. (D) An EMSA showing EBNA1 binding to a positive-control oligonucleotide (EBV Rep* site 1; lanes 1 to 3), oligonucleotides that include c-Myc site 1 (lanes 4 to 6) or c-Myc site 2 (lanes 7 to 9), and a negative-control oligonucleotide (a portion of the EBV Raji origin; lanes 10 to 12). For each oligonucleotide, EBNA1 amounts added were none in the left lanes, 1.5 μg EBNA1 in the middle lanes, and 6 μg in the right lanes (*n* = 3; a representative image is shown). (E) Chromatin immunoprecipitation (ChIP)-qPCR measurement of EBNA1 binding to the sites identified in panel C relative to EBNA1 binding to a negative-control region in the genome (proximal to the rhodopsin gene, where there are no predicted EBNA1-binding sites). Data shown are relative to IgG binding at each of the sites (*n* = 5; error bars show standard error). *, *P* < 0.05.

EBV is unique in its ability to infect and efficiently drive resting B cells to proliferate. It does so in part by increasing the expression of the c-Myc proto-oncogene. This increase in c-Myc levels is perilous, though, because it can also lead to the triggering of apoptotic signals (54) and in some cases increased expression of immune ligands (55). Modulating c-Myc levels could be key to balancing proliferation versus reducing apoptosis and expression of immune cell ligands, such as ULBP1. We have now found that EBV uses EBNA1 to limit c-Myc’s expression in newly infected peripheral B cells. We infected B cells with wt-EBV and ΔEBNA1-EBV and found that by 4 days postinfection, c-Myc mRNA levels are 60% higher in cells infected with ΔEBNA1-EBV (Fig. 5A). We confirmed EBNA1’s repression of c-Myc in H1299 cells expressing EBNA1 (Fig. 5B). Thus, while EBV induces the expression of c-Myc in newly infected primary B cells to drive proliferation, EBNA1 caps this induction of c-Myc expression. To test whether EBNA1 regulates c-Myc expression directly, we used the PWM noted above (Fig. S1) to search for potential EBNA1-binding sites within the promoter region of the c-Myc gene. We identified two putative EBNA1-binding sites upstream of c-Myc’s transcriptional start site (Fig. 5C). One of these sites (c-Myc site 2) was calculated to have a position *P* value of 6.7e−6, placing its affinity among those of the cellular binding sites we have established to be bound by EBNA1 at physiological levels (27). EMSAs as well as ChIP followed by qPCR in the EBV-positive LCL 721 showed that EBNA1 binds the c-Myc promoter at both the predicted sites at significantly higher levels than at a genomic negative-control site (Fig. 5D and E). Taken together, these findings show that EBNA1 binds the c-Myc promoter region in latently infected cells and limits expression of this oncogene in newly infected B cells.

**FIG 6:**
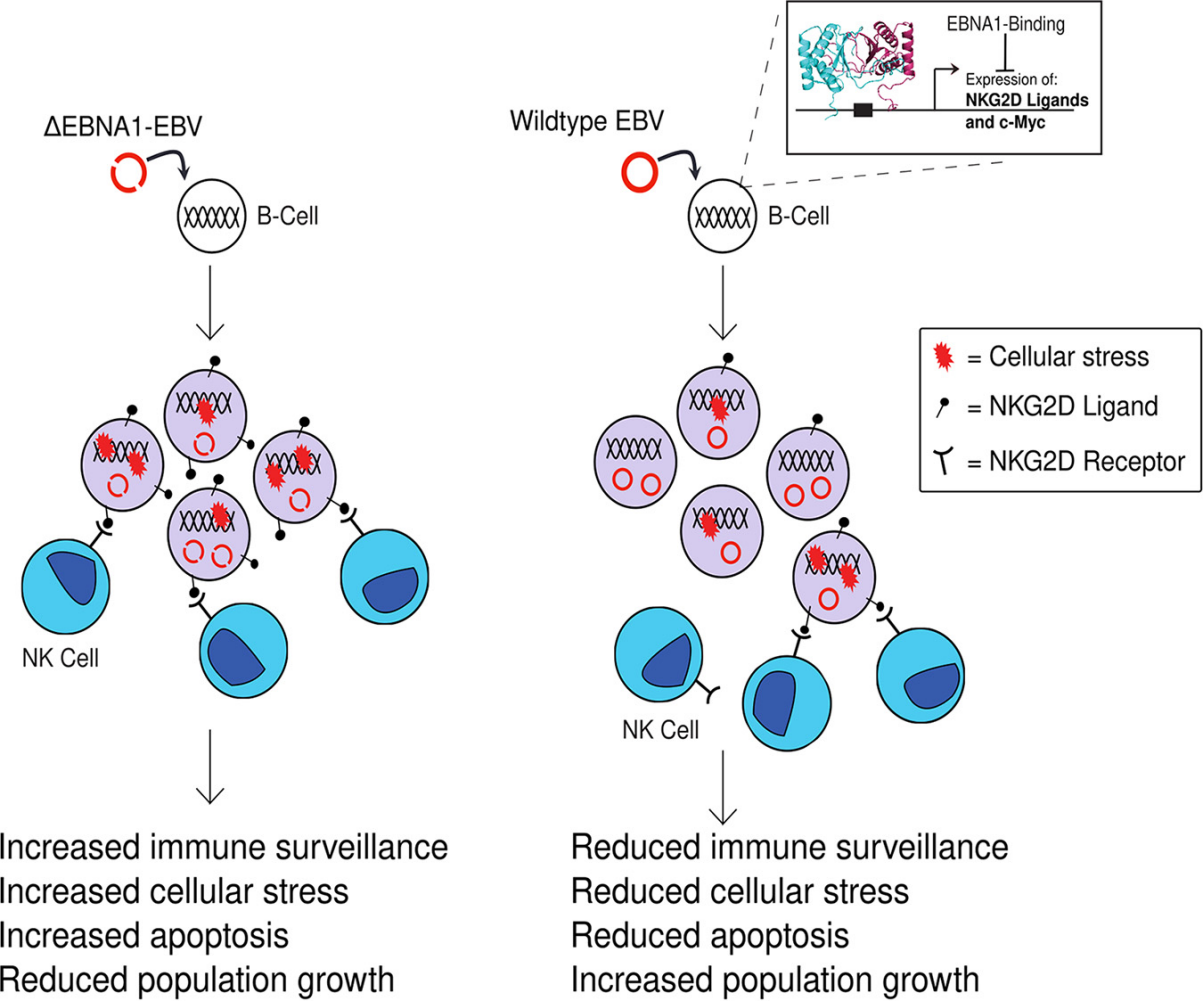
EBNA1 suppresses host immune response and enhances survival after infection. A model outlining how EBNA1 binds to genomic sites close to the transcriptional start site of NKG2D ligands ULBP1 and ULBP5 as well as the c-Myc gene and inhibits their expression. Inhibition of NKG2D ligands makes EBV-infected cells less susceptible to NK cell-mediated killing. Inhibition of c-Myc expression limits cellular stress responses and reduces apoptosis of newly infected cells. Limiting cellular stress can also reduce expression of immune cell ligands, further enhancing EBNA1-mediated immune evasion and survival of cells newly infected with EBV.

In summary, we show that in B cells newly infected with EBV, EBNA1 downregulates the NKG2D ligand ULBP1 by directly binding near its TSS. EBNA1 can also directly downregulate other NKG2D ligands, such as ULBP5. Downregulating NKG2D ligands makes EBV-infected cells less susceptible to NK cell-mediated killing. In addition to directly reducing levels of NKG2D ligands, EBNA1 downregulates cellular responses to stress and/or DNA damage—as measured by γH2AX—after EBV infection of B cells and reduces apoptosis in newly infected cells. EBNA1 does so, at least in part, by binding to the promoter region of the c-Myc gene and capping its expression in newly infected peripheral B cells. Thus, we show that EBNA1 can suppress the host innate immune response through multiple pathways to increase the survival of newly infected cells (Fig. 6).

## DISCUSSION

Infection with EBV elicits strong adaptive and innate immune responses (56, 57). For example, primary infection with EBV can lead to a massive expansion of viral antigen-specific CD8^+^ T cells (14) and the accumulation of specific subtypes of natural killer (NK) cells in the tonsillar area (15), typically the site of initial EBV infection. However, some B cells newly infected with EBV manage to slip past host immune surveillance and establish a lifelong latent infection in more than 95% of the world’s adult population (12). We have examined this immune evasion with a derivative of EBV that lacks the key latent protein EBNA1. EBV, in part through EBNA1, subtly regulates components of the innate immune system and cellular stress response, ensuring that some newly infected B cells evade the immune system and survive to persist for the life of the host.

Insightful studies have established that the NKG2D receptor expressed on NK cells and CD8^+^ T cells helps to control EBV infections (58, 59). Individuals who have null mutations in the magnesium transporter 1 (*MAGT1*) gene have NK cells that express severely reduced levels of NKG2D and are both susceptible to uncontrolled chronic EBV infection and more likely to develop EBV-related malignancies (58). We show that EBNA1 directly downregulates the NKG2D ligands ULBP1 and ULBP5. Importantly, the downregulation of ULBP1—and potentially also ULBP5—has vital functional consequences for EBV-infected cells as higher levels of the NKG2D ligand lead to significantly increased recognition and killing of EBV-positive cells by NK cells. It has also been found that overexpression of the EBV microRNA (miRNA) MiR-BART2-5p reduced expression of the NKG2D ligand MICB in RKO colon carcinoma cells, and a sponge directed against this miRNA led to an increase in MICB levels in the EBV-positive LCL 721, rendering these cells more susceptible to NK cell-mediated killing (23). Another study (24) showed that B cells infected with EBV lacking the latent protein LMP2A expressed significantly higher levels of the NKG2D ligand ULBP4 than did B cells infected with wt-EBV. The B cells expressing higher levels of ULBP4 were killed more efficiently by CD8^+^ T cells, but the activity of NK cells was not explored. That LMP2A can regulate expression of ULBP4 could explain why in our study NOKs cells infected with Akata-EBV showed downregulation of both ULBP4 and ULBP5, but H1299 cells expressing EBNA1 downregulated only ULBP5; NOKs-Akata cells express both EBNA1 and LMP2A (52). Unsurprisingly, EBV seems to have evolved multiple pathways to regulate the host immune response, especially in the first days after infection (20, 25). For example, Nikitin et al. (19) observed an increase of about 1.5-fold in the number of cells positive for γH2AX foci when B cells were infected with EBNA3C-null EBV compared to infections with wt-EBV. The B cells we infect with EBNA1-null EBV still express EBNA3C (to 60% of wt levels at day 4 postinfection), yet we see close to a 3-fold increase in cells positive for γH2AX foci when B cells are infected with EBNA1-null EBV compared to infections with wt-EBV. We reason that this finding strongly supports a role for EBNA1 (likely in cooperation with EBNA3C) in attenuating activation of γH2AX postinfection. However, unlike LMP2A and EBNA3C, EBNA1 is expressed by all EBV-positive cells (60). EBNA1’s presence in all EBV-positive cells, both early and late after infection, further highlights the importance of EBNA1’s ability to directly downregulate NKG2D ligands and assist EBV-positive cells in continuing to evade immune surveillance.

In addition to avoiding detection by the immune system, B cells newly infected with EBV must balance proliferation against cellular stress and apoptosis. Infection with EBV induces naive B cells to enter the cell cycle and transition from G_0_ to G_1_ (61) by upregulating the expression of cellular genes, such as the oncoprotein c-Myc (62), as well as EBV’s transforming genes, including EBNA1 (13). However, the robust cellular proliferation that EBV induces, in part via c-Myc expression, also activates cellular stress responses (16, 17, 19), which can upregulate expression of NKG2D ligands (7), again raising the specter of immune detection and death of these infected cells. We show that B cells infected with ΔEBNA1-EBV have higher levels of c-Myc and apoptosis and show higher numbers of γH2AX-positive cells than B cells infected with wt-EBV, indicating that EBNA1 plays an important role in both limiting c-Myc expression and dampening cellular stress responses and apoptosis, increasing the likelihood that some EBV-infected cells survive and evade the immune system. Levels of c-Myc mRNA in H1299 cells expressing EBNA1 were less than 33% of c-Myc levels in H1299 cells not expressing EBNA1. This difference in c-Myc levels with and without EBNA1 is significant at the steady-state level as cells often need to regulate levels of c-Myc precisely to avoid death and continue to proliferate (63).

Cellular γH2AX is most widely known as a marker for DNA damage, but γH2AX foci can develop even in the absence of a DNA damage response. For example, serum starvation—which is not known to cause DNA strand breaks—leads to the formation of γH2AX foci in HaCaT cells and mouse embryonic fibroblasts in a p38-dependent manner (64). Others have shown cell cycle-dependent phosphorylation of γH2AX in a CHK2/DNA-PKcs-dependent manner even in the absence of DNA damage (65). In the context of EBV infection of B cells, several reports have observed increased levels of γH2AX in B cells postinfection, but whether the ATR/Chk1 or the ATM/Chk2 pathway leads to the activation of H2AX is controversial. Nikitin et al. (19) observed activation of ATM/Chk2 after EBV infection of peripheral B cells with EBV, but Mordasini et al. (16) observed only ATR/Chk1 activation after infection of peripheral or tonsillar B cells with EBV. Koganti et al. (18) showed ATR activation and STAT3-mediated interruption of the ATR/Chk1 signaling axis after EBV infection of primary B cells. More recently, Pich et al. (17) tested small-molecule inhibitors of ATM and ATR, kinases that ultimately activate γH2AX in response to different stresses. They showed that a small-molecule inhibitor of ATM, which is activated primarily in response to DNA damage, did not reduce the fluorescent intensity of γH2AX foci in EBV-infected B cells. In contrast, a small-molecule inhibitor of ATR, which is activated in response to persistent single-stranded DNA, especially at stalled replication forks during DNA replication stress, reduced γH2AX levels in EBV-infected B cells. We confirmed that the downstream measure of either the ATR/Chk1 or the ATM/Chk2 pathway being activated, i.e., increased levels of γH2AX, was significantly lower in B cells infected with wt-EBV compared to γH2AX levels in B cells infected with ΔEBNA1-EBV, strongly indicating that the presence of EBNA1 reduces cellular stress responses in EBV-infected B cells. Future experiments can tease out which pathways are activated when B cells are infected with ΔEBNA1-EBV.

Some previous research on the relationship between EBNA1, c-Myc, and stress responses in B cells newly infected with EBV seemed to indicate that EBNA1 is dispensable for the initial survival and proliferation of the infected cells (17). The differences between these prior findings and our results likely reflect our use of peripheral B cells and the use of adenoidal B cells by Pich et al. (17). There can be significant differences in the growth and survival dynamics of adenoidal versus peripheral B cells infected with EBV, with adenoidal B cells typically showing faster growth and less death after EBV infection (66). Additionally, Pich et al. (17) examined different characteristics—activation, cell cycle entry, and proliferation—of newly infected B cells compared to our assays testing apoptosis and cellular stress response in the newly infected cells.

Extensive evidence exists showing EBNA1 can bind cellular DNA and regulate cellular gene expression both positively and negatively (reviewed in the work of Wilson et al. [67]). However, the mechanisms by which EBNA1 modulates cellular transcription are not completely clear. EBNA1 can mediate DNA looping of both viral and cellular DNAs (68, 69), and EBV-negative cells transfected with EBNA1 or engineered to express derivatives of EBNA1 develop more regions of open chromatin (70), either of which could be mechanisms by which EBNA1 affects transcription. Genome-wide assays for EBNA1 binding and transcription modulation have shown EBNA1 binding to chromatin with both active and repressive marks (36, 71), which could indicate that EBNA1 also serves as a cofactor for cellular proteins that activate or repress transcription. Indeed, EBNA1 is known to interact with several such proteins, including P32/TAP (72), nucleolin (73), nucleosome assembly factor 1 (NAP1), and template activator factor I (TAF-I) (74) among others. While we do not yet know exactly how EBNA1 downregulates the expression of NKG2D ligands or c-Myc in newly infected B cells, establishing its direct role at these genomic locations allows future experiments to elucidate how EBNA1 suppresses transcription.

Importantly, our experiments were carried out primarily in B cells newly infected with wt-EBV or ΔEBNA1-EBV and not in LCLs. LCLs have served as a powerful *in vitro* model of EBV infection and persistence but are selected for their growth in the absence of any immune selection. By focusing on the first few days postinfection, we have avoided this potential bias and been able to unearth how the EBV oncoprotein EBNA1—in addition to being indispensable for the maintenance of the viral genome in latently infected cells—contributes unexpectedly to cells newly infected with EBV by limiting their immune detection and balancing proliferation and stress responses to ultimately establish lifelong infection in hosts.

## MATERIALS AND METHODS

### Cell lines.

Daudi (75), 721 (76), H1299 (77), HEK293 (78), K562 (79), and NOKs and NOKs-Akata (80) cells have been described previously. Additional details are given in Text S1 in the supplemental material.

10.1128/mBio.02243-21.1 TEXT S1 Supplemental methods. Download Text S1, DOCX file, 0.02 MB. Copyright © 2021 Westhoff Smith et al. 2021 Westhoff Smith et al. https://creativecommons.org/licenses/by/4.0/ This content is distributed under the terms of the Creative Commons Attribution 4.0 International license.

### Analysis of RNA-seq data.

We used publicly available data derived from http://ebv-b.helmholtz-muenchen.de (42) to analyze the expression of NKG2D ligands before and 2, 4, and 8 days after EBV infection of B cells *in vitro*. Genes with an average expression lower than 25 normalized counts were excluded from further analyses.

### Predicting EBNA1 binding.

The 16-nucleotide PWM for the binding site of EBNA1 was created using the online software MEME (http://meme.nbcr.net/meme/) with the sequences of 73 identified EBNA1-binding sites (27). Additional details are given in Text S1.

### EMSAs.

EMSAs were carried out using IR700-labeled oligonucleotides ordered from Integrated DNA Technologies (IDT) and recombinant EBNA1 protein from Abcam (ab138345). Labeled oligonucleotides were dimerized by dilution to 20 pmol/μl in 1× Tris-EDTA (TE), heating to 100°C for 5 min, and cooling slowly to room temperature. Indicated amounts of the EBNA1 protein and 1 μl of a 1:100 dilution of dimerized oligonucleotides was used for the EMSAs. Five percent polyacrylamide gels were used for electrophoresis, and gels were visualized using a Li-Cor Odyssey imager. More details, including sequences of the oligonucleotides used (Table S3), are provided in the supplemental material.

10.1128/mBio.02243-21.9 TABLE S3 Oligonucleotides used in this study for EMSAs. Download Table S3, DOCX file, 0.01 MB. Copyright © 2021 Westhoff Smith et al. 2021 Westhoff Smith et al. https://creativecommons.org/licenses/by/4.0/ This content is distributed under the terms of the Creative Commons Attribution 4.0 International license.

### Chromatin immunoprecipitation.

Cells were cross-linked in 1% methanol-free formaldehyde, quenched with 0.5 M glycine, washed twice with ice-cold phosphate-buffered saline (PBS), and then lysed in the presence of a protease inhibitor cocktail (Roche). Chromatin was sheared to fragments 300 to 500 bp in size, and sonicated chromatin from 5 × 10^6^ cell equivalents was precleared and incubated with either anti-EBNA1 (81) or isotype-matched IgG overnight, after which protein A/G beads (Pierce) conjugated with rabbit anti-rat antibody were added. Samples were then placed on a magnetic rack and washed serially. Antibody-bound chromatin was then eluted from the magnetic beads, and cross-links were reversed. Chromatin was treated with RNase and extracted by phenol, chloroform, and Qiagen PCR cleanup Kits. Isolated chromatin was analyzed by qPCR with primer and probe sets listed in Table S1. Additional details are given in Text S1.

10.1128/mBio.02243-21.7 TABLE S1 Primers used in this study for ChIP. Download Table S1, DOCX file, 0.02 MB. Copyright © 2021 Westhoff Smith et al. 2021 Westhoff Smith et al. https://creativecommons.org/licenses/by/4.0/ This content is distributed under the terms of the Creative Commons Attribution 4.0 International license.

### Primary B-cell isolation.

Peripheral blood mononuclear cells (PBMCs) were isolated from whole blood or buffy coat (Interstate Blood Bank Inc.) by centrifugation over a cushion of Ficoll-Paque. Platelets were removed from PBMCs by repeated washes with phosphate-buffered saline (PBS)–EDTA–fetal bovine serum (FBS). B cells were negatively isolated from PBMCs using the magnetically activated cell sorting (MACS) B-cell isolation kit II (Miltenyi Biotec). Natural killer cells were positively selected using MACS CD56 microbeads (Miltenyi Biotec).

### EBER *in situ* hybridization.

Cells were washed in 1× PBS, spread onto slides, fixed with 4% paraformaldehyde, and then permeabilized with 0.5% Triton X-100. *In situ* hybridization was carried out as described previously (82) with the use of biotinylated 30-mer oligonucleotides for EBER detection (PanPath PLB101). Hybridized probe was detected with a solution containing streptavidin conjugated to Cy3 (Cytocell). Cellular DNA was counterstained with mounting medium containing 4′,6-diamidino-2-phenylindole (DAPI) (Vector). Cells were imaged using a Zeiss ApoTome.

### RNA isolation, reverse transcription, and real-time qPCR (RT-qPCR).

Total RNA was isolated using either the RNeasy MiniPrep Kit (Qiagen) or the Direct-zol RNA purification kit (Zymo) and included a DNase treatment according to manufacturer’s instructions.

After isolation, 1 to 2 μg of total RNA was reverse transcribed using the Applied Biosystems high-capacity reverse transcription kit with MultiScribe murine leukemia virus (MuLV) reverse transcriptase. Instead of the supplied random primer, 5 μM 20-mer oligo(dT) was used. RT-qPCR was carried out as previously described (83). Primary B-cell and H1299 mRNA levels were normalized to TATA box-binding protein (TBP) and glyceraldehyde-3-phosphate dehydrogenase (GAPDH), respectively, and fold enrichment was determined using the threshold cycle (ΔΔ*C_T_*) method (84). Primers and probes used are listed in Table S2.

10.1128/mBio.02243-21.8 TABLE S2 Primers used in this study for mRNA detection. Download Table S2, DOCX file, 0.02 MB. Copyright © 2021 Westhoff Smith et al. 2021 Westhoff Smith et al. https://creativecommons.org/licenses/by/4.0/ This content is distributed under the terms of the Creative Commons Attribution 4.0 International license.

### Natural killer cell degranulation assay.

Primary NK cells were treated with IL-2 (10 ng/ml BioLegend) overnight and plated at an effector-to-target ratio of 10:1 with autologous untreated B cells or autologous B cells that had been infected 4 days prior with wt-EBV or ΔEBNA1-EBV. As a positive control, NK cells were also cultured with K562 cells, which lack MHC class I and II molecules but retain NKG2D ligand expression. For antibody blocking experiments, NK cells were treated with 50 μg/ml NKG2D blocking antibody (R&D Systems MAB139) for 30 min at room temperature prior to cultivation with target cells. NK cell degranulation assays were performed as previously described (48) with the antibodies CD56-allophycocyanin (APC) (BioLegend), CD107a-phycoerythrin (PE) (BioLegend), and CD19-fluorescein isothiocyanate (FITC) (BioLegend). Flow cytometric analysis of live cells was performed on a FACSAria III equipped with 403-nm, 488-nm, 561-nm, and 637-nm lasers. A total of 30,000 to 60,000 events was acquired and analyzed using FlowJo software. The analysis was performed on gated cells that were characteristic of the lymphocyte population. This population was then gated for CD19^−^ CD56^+^ NK cells. Within this population the expression of CD107a was determined.

### Generating mutants using CRISPR-Cas9.

The CRISPR Design tool (crispr.mit.edu) was used to identify potential target sequences at EBNA1-binding sites. Spacers were synthesized by Integrated DNA Technologies (Coralville, IA), phosphorylated and annealed, and then cloned into pSpCas9(BB)-2A-GFP (PX458) (85), a gift from Feng Zhang (Addgene plasmid no. 48138). Constructs were verified by Sanger sequencing and transfected into H1299 cells with Lipofectamine 3000 (Life Technologies). Sequences targeted by guide RNAs near the ULBP5 TSS were amplified by PCR, cloned into PCR-Blunt (Invitrogen), and verified by sequencing.

### Immunofluorescence staining.

B cells were spread on a microscope slide, fixed in 4% paraformaldehyde (PFA), and then permeabilized with 0.5% Tween 20 and blocked with 5% normal goat serum. Additional details and antibodies used are listed in Text S1.

### Apologix apoptosis detection.

The Apologix assay for detection of apoptosis was carried out as previously described (86). Briefly, cells were stained with SR-VAD-FMK (SR100; Cell Technology Inc) or FAM-VAD-FMK (FAM100; Cell Technology Inc) following the manufacturer’s protocol with two alterations. First, the cells were stained at an 0.5× final concentration of SR-VAD-FMK or FAM-VAD-FMK rather than at a 1× final concentration. Second, cells were washed in 1× PBS rather than the manufacturer’s supplied wash buffer. After staining, cells were fixed using a 1× final concentration of fixative solution supplied by the manufacturer. Cells were analyzed and counted on an inverted fluorescence microscope (Axiovert 200M; Zeiss) using the Texas Red and FITC filter sets.

### Statistics.

Microsoft Excel and Mstat 5.5 were used for statistical analysis. All statistics reported are the results of a two-sided Student *t* test, unless otherwise stated.
