# Supplementary material for: The Epstein-Barr Virus Oncogene EBNA1 Suppresses Natural Killer Cell Responses and Apoptosis Early after Infection of Peripheral B Cells
Source: mBio. 2021 Nov 16;12(6):e02243-21. doi: 10.1128/mBio.02243-21 (PMC8593684; doi:10.1128/mBio.02243-21)
Supplement: FIG S1 [file mbio.02243-21-sf001.docx]

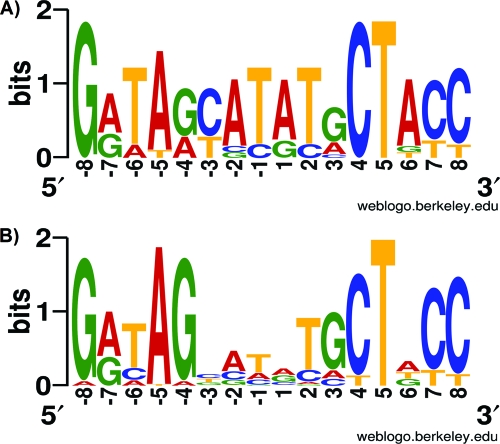


**Figure 1.** EBNA1 PWM used to predict EBNA1 binding sites near ULBP1, ULBP4, ULBP5 and c-Myc transcription start sites. We used sequences of 73 previously identified EBNA1-binding sites^1^ to generate a 16-nucleotide PWM for the binding site of EBNA1 with the online software MEME (<http://meme.nbcr.net/meme/>).
