# Supplementary material for: The Epstein-Barr Virus Oncogene EBNA1 Suppresses Natural Killer Cell Responses and Apoptosis Early after Infection of Peripheral B Cells
Source: mBio. 2021 Nov 16;12(6):e02243-21. doi: 10.1128/mBio.02243-21 (PMC8593684; doi:10.1128/mBio.02243-21)
Supplement: FIG S2A [file mbio.02243-21-sf002a.docx]

**Figure 2A.** At different time points post-infection, we compared mRNA levels of the EBV latent genes LMP1, EBNA2 and EBNA3C by qRT-PCR in cells infected with either ΔEBNA1-EBV or wt-EBV. Levels of the EBV mRNAs were normalized to those of TATA-box binding protein in those cells. The chart shows a ratio of the levels of different EBV mRNAs in cells infected with ΔEBNA1-EBV compared to levels of those mRNAs in cells infected with wt-EBV (n = 3; error bars show standard deviation). *P < 0.05.
