# Supplementary material for: The Epstein-Barr Virus Oncogene EBNA1 Suppresses Natural Killer Cell Responses and Apoptosis Early after Infection of Peripheral B Cells
Source: mBio. 2021 Nov 16;12(6):e02243-21. doi: 10.1128/mBio.02243-21 (PMC8593684; doi:10.1128/mBio.02243-21)
Supplement: FIG S2B [file mbio.02243-21-sf002b.docx]

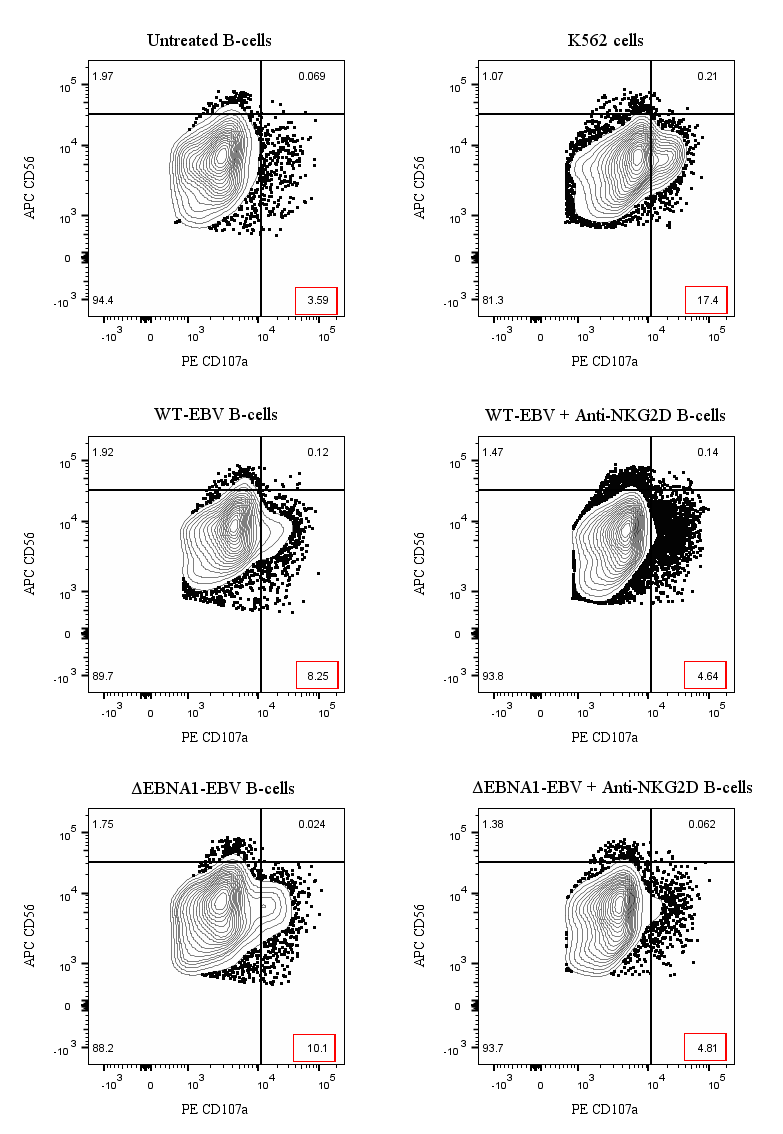


**Figure 2B.** Representative NK cell assay flow cytometry data presenting contour plots of CD107A-positive NK cells that had been co-cultured with the target cells as labeled. The percentage of cells within the target quadrant is outlined in red.
