# Supplementary material for: The Epstein-Barr Virus Oncogene EBNA1 Suppresses Natural Killer Cell Responses and Apoptosis Early after Infection of Peripheral B Cells
Source: mBio. 2021 Nov 16;12(6):e02243-21. doi: 10.1128/mBio.02243-21 (PMC8593684; doi:10.1128/mBio.02243-21)
Supplement: FIG S3A [file mbio.02243-21-sf003a.docx]

**Figure 3A:** Expression of ULBP4 and UBLP5 mRNA in NOKs cells in the presence or absence of EBV quantified by qRT-PCR and normalized to GAPDH expression (n = 3; error bars show standard error).
