# Supplementary material for: The Epstein-Barr Virus Oncogene EBNA1 Suppresses Natural Killer Cell Responses and Apoptosis Early after Infection of Peripheral B Cells
Source: mBio. 2021 Nov 16;12(6):e02243-21. doi: 10.1128/mBio.02243-21 (PMC8593684; doi:10.1128/mBio.02243-21)
Supplement: FIG S3B [file mbio.02243-21-sf003b.docx]

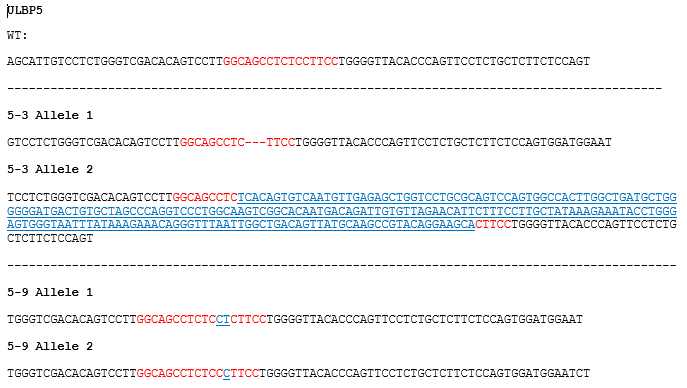


**Figure 3B.** Mutations in the EBNA1 binding site near the ULBP5 transcription start site introduced by CRISPR/Cas9 mutagenesis. Sequences from H1299 clones – 5-3 and 5-9 – are shown here. The sequence in red shows the EBNA1 binding site; red dashes (-) indicate deletions and blue text indicates insertions.
