# Supplementary material for: The Epstein-Barr Virus Oncogene EBNA1 Suppresses Natural Killer Cell Responses and Apoptosis Early after Infection of Peripheral B Cells
Source: mBio. 2021 Nov 16;12(6):e02243-21. doi: 10.1128/mBio.02243-21 (PMC8593684; doi:10.1128/mBio.02243-21)
Supplement: TEXT S1 [file mbio.02243-21-s0001.docx]

**Supplemental Data**

**Supplemental Methods**

**Cell lines**

Primary B-cells, EBV-positive 721 cells and EBV-negative Daudi cells were grown in RPMI 1640 (Gibco) supplemented with 10% fetal bovine serum (FBS). HEK293 and H1299 cells were grown in Dulbecco’s modified Eagle medium or DMEM (Gibco) supplemented with 10% FBS. NOKs and NOKs-EBV clones derived by infection with the Akata strain of EBV, selection in G418, and cloning, were maintained in Keratinocyte Serum-Free media supplemented with human epidermal growth factor and bovine pituitary extract (Life Technologies). EBNA1-positive HEK293 cells were engineered by stable infections with retroviral constructs expressing EBNA1 or derivatives of EBNA1. The HEK293 ΔEBNA1-EBV producer cells^1^ were induced to produce virus by treatment with 12-O-tetradecanoyl-phorbol-13-acetate (TPA) and sodium butyrate. HEK293 cells carrying 2089 wt-EBV^2^ and stably expressing tamoxifen-inducible EBV Zta activator of lytic gene expression were induced to produce virus by treatment with 200nM 4-hydroxy-tamoxifen (Sigma). H1299 cells transfected with the EBV-derived plasmid p220.2^3^ were grown in the presence of 200ug/ml hygromycin. All cell culture media were supplemented with 200U/mL penicillin and 200ug/mL streptomycin. All cells were grown at 37°C in a 5% CO2 humidified atmosphere. Viral titers were determined using EBV-negative Daudi cells.

**EBNA1 Binding-Prediction**

We developed a program to identify DNA sequences similar to the binding sites of EBNA1, as defined by a position-weighted matrix (PWM), within a fixed search sequence. A PWM defines the DNA motif to which a protein binds by determining the probability of a particular nucleotide in each position in a binding sequence. We used sequences of 73 previously identified EBNA1-binding sites^4^ to generate a 16-nucleotide PWM for the binding site of EBNA1 with the online software MEME (<http://meme.nbcr.net/meme/>). Each position along the search sequence, consisting of that of the human genome or that of EBV, was compared to the fixed search sequence for both the Watson and Crick strands; a complete match of the nucleotides in the PWM was considered a hit. Then, the PWM match p-values were determined for each 16-mer nearby, and p-values were generated by calculating the total number of possible 16-mers with higher match scores than the match score derived from the PWM for the given 16-mer. If a p-value for the EBNA1 PWM near the fixed search "hit" was below a threshold, we defined the hit as a binding site for EBNA1.

**EMSA**

Duplex IR700-labeled oligonucleotides were added to EMSA buffer (2.5mM DTT; 0.25% Tween-20; 50ng/ul poly(dI.dC); 20mM HEPES; 40mM KCL; 1mM MgCl_2_; 1mM EDTA; 10% glycerol) and indicated amounts of EBNA1 in a total volume of 20ul. The mixture was incubated in the dark at room temperature for 30 minutes. 5% TBE-polyacrylamide gels were used to run the EMSA assays at 70V for about 1 hour at room temperature. Gels were directly imaged using a LICOR Odyssey imager.

**Immunofluorescence staining**

Approximately 3 × 10^5^ B-cells were pelleted, washed in PBS, resuspended in 25 μl of PBS, spread on a microscope slide and dried at 37°C for 20 minutes then fixed in 4% PFA in PBS for 15 minutes, permeabilized in PBS containing 0.5% Tween-20 for 20 minutes then blocked in PBS with 0.2% Tween-20 containing 5% normal goat serum for 1 hour. A primary rabbit antibody, Anti-Phospho-Histone H2A.X (Ser139) (Cell Signaling #2577), was diluted 1:50. A secondary goat antibody, Anti-Rabbit Alexafluor 594 (Invitrogen Molecular Probes A11072), was diluted 1:100. Slides were mounted in Vectashield containing DAPI (Vector Laboratories). At least 50 cells were counted per condition.

**ChIP**

5x10^7 cells were cross-linked for 10 minutes in 1% methanol-free formaldehyde and quenched with 0.5 M glycine for 5 minutes. Cells were washed twice with ice-cold PBS then lysed in serial incubations for 10 minutes at 4 °C with Lysis Buffer I (50mM HEPES-KOH pH 7.5, 140mM NaCl, 1mM EDTA, 10% Glycerol, 0.5% NP-40, 0.25% Triton X-100), Lysis Buffer II (10mM Tris-HCL pH 8.0, 100mM NaCl, 1mM EDTA, 0.5mM EGTA, 0.1% Na-Deoxycholate, 0.5% N-lauroylsarcosine) and Lysis Buffer III (10mM Tris-HLC pH 8.0, 200mM NaCl, 1mM EDTA, 0.5mM EGTA) in the presence of a protease inhibitor cocktail (Roche). Chromatin was sheared to fragments 300 to 500bp in size by eighteen 20-second pulses at setting 3 on a Fisher Scientific Sonic Dismembranator Model 100 equipped with a microtip probe. Sonicated chromatin from 5x10^6 cell equivalents was incubated overnight at 4 °C with 8ug Rat IgG2a (Biolegend Clone RTK2758); then precleared for 1 hour at room temperature with Protein A/G magnetic beads (Pierce) that had been washed and resuspended in Washing Buffer (25mM Tris, 150mM NaCl, 0.05% Tween-20); then incubated overnight at 4 °C with 24ug Rabbit Anti-Rat IgG (Jackson Immunoresearch 312-005-045). Precleared chromatin was then incubated with 8ug Anti-EBNA1 ^10^ or overnight at 4 °C after which Protein A/G beads conjugated with Rabbit Anti-Rat antibody were added for 1 hour at room temperature. Samples were then placed on a magnetic rack and washed serially with Wash Buffer I (50mM HEPES pH 7.5, 140 mM NaCl, 1% Triton X-100, 0.1% deoxycholate, protease inhibitors), Wash Buffer II (50mM HEPES pH 7.5, 500mM NaCl, 1% Triton X-100, 0.1% deoxycholate,), Wash Buffer III (10mM Tris pH 8.0, 250 mM LiCl, 0.5% NP-40, 0.5% deoxycholate, 1mM EDTA) and Tris-EDTA. Antibody bound chromatin was then eluted from the magnetic beads with Elution Buffer (50mM Tris pH 8.0, 1% SDS, 10mM EDTA) in a 65°C water bath. The protein crosslink was reversed by overnight incubation at 65°C with 200mM NaCl, 0.5% SDS and 200ug/mL Proteinase K. Chromatin was treated with RNase and extracted by phenol, chloroform, and a Qiagen PCR Clean-Up kit. Isolated chromatin was analyzed by q-PCR with Primer and Probe sets listed in Table S3. Fold enrichment was determined after a two-step normalization with mock IgG pull down samples and PCR against a non-specific genomic site (Rhodopsin).

**Western blotting**

Cells were resuspended in 1x NET lysis buffer (20mM Tris pH 7.5, 150 mM NaCl, 1mM EDTA, 0.5% Triton X-100) and then lysed by adding an equal volume of 2x sample buffer (20 mM Tris pH 6.5, 100mM NaCl, 10% glycerol, 6% SDS, 5% beta-mercaptoethanol, 0.04% bromophenol blue). Samples were briefly sonicated. Cell lysates (1-2 x 10^5 per sample) were then separated on 10% SDS-PAGE gels and transferred electrophoretically to nitrocellulose membranes. The blots were blocked in blotto (5% nonfat milk, 0.05% Tween-20 in 1x PBS) overnight and probed with primary antibodies followed by alkaline phosphatase-labeled secondary antibodies. Rat monoclonal 1H4 was used at 1:50 dilution to detect EBNA1. Goat polyclonal Actin antibody (Santa Cruz Biotechnology) was used at a 1:500 dilution. Alkaline phosphatase was visualized using 5-bromo-4-chloro-3’-indolyl phosphate p-toluidine salt and nitroblue tetrazolium chloride.
