# Supplementary material for: The Epstein-Barr Virus Oncogene EBNA1 Suppresses Natural Killer Cell Responses and Apoptosis Early after Infection of Peripheral B Cells
Source: mBio. 2021 Nov 16;12(6):e02243-21. doi: 10.1128/mBio.02243-21 (PMC8593684; doi:10.1128/mBio.02243-21)
Supplement: TABLE S3 [file mbio.02243-21-st003.docx]

**Table S3 Oligonucleotides used in this study for EMSAs**

Oligo Sequence

| ULBP1 1 | CCCTTTTGGAAGCATCTGCTTAAACCC |
| --- | --- |
|  | GGGTTTAAGCAGATGCTTCCAAAAGGG |
|  |  |
| ULBP1 2 | CCCTTTTAACAGCATTTCCTACCAAACCC |
|  | GGGTTTGGTAGGAAATGCTGTTAAAAGGG |
|  |  |
| c-Myc 1 | CCCTTTTGGTAACCTCTCCTTCCAAACCC |
|  | GGGTTTGGAAGGAGAGGTTACCAAAAGGG |
|  |  |
| c-Myc 2 | CCCTTTTGACAGTATGAGCTTCTAAACCC |
|  | GGGTTTAGAAGCTCATACTGTCAAAAGGG |
|  |  |
| Positive | CCCTTTTGGAAATACGTCCTACCAAACCC |
|  | GGGTTTGGTAGGACGTATTTCCAAAAGGG |
|  |  |
| Negative | CCCTTTTGGAAAGGCCTGCTGTCAAACCC |
|  | GGGTTTGACAGCAGGCCTTTCCAAAAGGG |
