# Supplementary material for: The Epstein-Barr Virus Oncogene EBNA1 Suppresses Natural Killer Cell Responses and Apoptosis Early after Infection of Peripheral B Cells
Source: mBio. 2021 Nov 16;12(6):e02243-21. doi: 10.1128/mBio.02243-21 (PMC8593684; doi:10.1128/mBio.02243-21)
Supplement: TABLE S1 [file mbio.02243-21-st001.docx]

**Table S1 Primers used in this study for ChIP**

Gene Primer Sequence

| MYC_1 | Probe | (56 FAM)ACCATCTTCCCATTCTTCCCTTCCT(3IABkFQ) |
| --- | --- | --- |
|  | Forward | TGACAGTATGAGCTTCTCCCTA |
|  | Reverse | TCGCAACTTTATAATCCCTCTGG |
|  |  |  |
| MYC_2 | Probe | (56 FAM)ACCTCTCCTTCCTTCCTTATTTCCCT(3IABkFQ) |
|  | Forward | CTCGATACTCATGGTGCCTTT |
|  | Reverse | TCTATTTCTCTGCTGCTGGTG |
|  |  |  |
| ULBP1_1 | Probe | (56 FAM)TTCCAAGTGCACTCAGGTTGATGGCA(3IABkFQ) |
|  | Forward | TGGTTTCTCTGCTCCCAGTT |
|  | Reverse | CACAGGTCCAAGGACTGGAT |
|  |  |  |
| ULBP1_2 | Probe | (56 FAM)TGGGAGCCCATTTCAGGAACAGCATT(3IABkFQ) |
|  | Forward | CTTCCTGCTGACAGTTGACG |
|  | Reverse | AGGAATGCCTGTTGCTGA |
|  |  |  |
| FR | Probe | (56 FAM)AAG ATC AAG GAG CGG GCA GTG AA(3IABkFQ) |
|  | Forward | AGA AGC AGG CGA AGA TTC AG |
|  | Reverse | CCA TTT TAG TCA CAA GGG CAG |
|  |  |  |
| Rhodopsin | Probe | (56 FAM)AGC CTC TAG TTT CCA GAA GCT GCA CA(3IABkFQ) |
|  | Forward | ATC AGG AAC CAT TGC CAC GTC CTA |
|  | Reverse | AGG CCA AAG ATG GAC ACA CAG AGT |
