# Supplementary material for: The Epstein-Barr Virus Oncogene EBNA1 Suppresses Natural Killer Cell Responses and Apoptosis Early after Infection of Peripheral B Cells
Source: mBio. 2021 Nov 16;12(6):e02243-21. doi: 10.1128/mBio.02243-21 (PMC8593684; doi:10.1128/mBio.02243-21)
Supplement: TABLE S2 [file mbio.02243-21-st002.docx]

**Table S2 Primers used in this study for mRNA detection**

Gene Primer Sequence

| c-Myc | Probe | (56 FAM)CCCCTCAACGTTAGCTTCACCAACA(3IABkFQ) |
| --- | --- | --- |
|  | Forward | TTCGGGTAGTGGAAAACCAG­­ |
|  | Reverse | AGTAGAAATACGGCTGCACC |
|  |  |  |
| ULBP1 | Probe | (56 FAM)AATAGGGAGTCTTTCCCACATTGCCC(3IABkFQ) |
|  | Forward | CACACCTACCGCCATCTAATC |
|  | Reverse | CTGCATATGGTTGGCCAGATA |
|  |  |  |
| ULBP4 | Probe | (56 FAM)ACCAGTGCACCGTTCTGCTTCA(3IABkFQ) |
|  | Forward | TCAAACCCCAGATAAAGACCAG |
|  | Reverse | TCCAGGTCATGTTCATTGCG |
|  |  |  |
| ULBP5 | Probe | (56 FAM)AAGCGGGAGGCGTAGAAGGAAC(3IABkFQ) |
|  | Forward | GCGATCCAACTCCCCAATG |
|  | Reverse | CTGAACTTAGGGATGACGGTG |
|  |  |  |
| TBP | Probe | (56 FAM)AGAGCCACGAACCACGGCACTGATTT(3IABkFQ) |
|  | Forward | TATAATCCCAAGCGGTTTGCTGCG |
|  | Reverse | TGCCAGTCTGGACTGTTCTTCACT |
|  |  |  |
| GAPDH | Probe | (56 FAM)TTCCTGGTATGACAACGAATTTGGCTACAGC(6-FAM) |
|  | Forward | TCAACGACCACTTTGTCAAGCT |
|  | Reverse | CCATGAGGTCCACCACCCT |
|  |  |  |
| EBNA1 | Probe | (56 FAM)TCCTCTGGAGCCTGACCTGTGATCG(3IABkFQ) |
|  | Forward | TGAAGACTAAGTCACAGGCTTAGC |
|  | Reverse | CATGATTCACACTTAAAGGAGACGG |
|  |  |  |
| EBNA2 | Probe | (56 FAM)CCAATACATGAACCGGAGTCCCATAATAGCC(3IABkFQ) |
|  | Forward | CGGCAACCCCTAACGTTTC |
|  | Reverse | GGGAAGAGAATGGGAGCCTC |
|  |  |  |
| EBNA3C | Probe | (56 FAM)CATTAATGCCACCACGCCAAAAAGGC(3IABkFQ) |
|  | Forward | CAAGGTGCATTTACCCCACTG |
|  | Reverse | GGGCAGGTCCGTGAGAACT |
|  |  |  |
| LMP1 | Probe | (56 FAM)TCATTCCCGTCGTGTTGCAATCCCAAGTACAG(3IABkFQ) |
|  | Forward | TTCTCTGTCCACTTGGAGCCCTTT |
|  | Reverse | AATGCCTGTCCGTGCAAATTCCAG |
